# Evaluation of Deep Neural Network Compression Methods for Edge Devices Using Weighted Score-Based Ranking Scheme

**DOI:** 10.3390/s21227529

**Published:** 2021-11-12

**Authors:** Olutosin Ajibola Ademola, Mairo Leier, Eduard Petlenkov

**Affiliations:** 1 Embedded AI Research Laboratory, Department of Computer Systems, Tallinn University of Technology, Ehitajate tee 5, 19086 Tallinn, Estonia; mairo.leier@taltech.ee; 2 Centre for Intelligent Systems, Department of Computer Systems, Tallinn University of Technology, Ehitajate tee 5, 19086 Tallinn, Estonia; eduard.petlenkov@taltech.ee

**Keywords:** deep neural network compression, compression method evaluation, weighted score-based ranking, embedded deep learning, edge computing

## Abstract

The demand for object detection capability in edge computing systems has surged. As such, the need for lightweight Convolutional Neural Network (CNN)-based object detection models has become a focal point. Current models are large in memory and deployment in edge devices is demanding. This shows that the models need to be optimized for the hardware without performance degradation. There exist several model compression methods; however, determining the most efficient method is of major concern. Our goal was to rank the performance of these methods using our application as a case study. We aimed to develop a real-time vehicle tracking system for cargo ships. To address this, we developed a weighted score-based ranking scheme that utilizes the model performance metrics. We demonstrated the effectiveness of this method by applying it on the baseline, compressed, and micro-CNN models trained on our dataset. The result showed that quantization is the most efficient compression method for the application, having the highest rank, with an average weighted score of 9.00, followed by binarization, having an average weighted score of 8.07. Our proposed method is extendable and can be used as a framework for the selection of suitable model compression methods for edge devices in different applications.

## 1. Introduction

In deep learning, object classification tasks are solved using Convolutional Neural Networks (CNNs). CNNs are variants of Deep Neural Network (DNN) architectures that accept batches of images as input and return the probability vectors of all the possible outcomes [1]. These architectures are used as the backbone of state-of-the-art DNN-based object detection methods. R-CNN [2] was one of the most successful methods proposed to solve object classification, localization, and segmentation problems. R-CNN used AlexNet (a variant of the CNN architecture developed in [1], having over 62M trainable parameters and requiring a storage size of 250MB) as the backbone of the network. Other CNN architectures used as the backbone of object detection models are ResNet-50 [3], which requires over 95MB of storage space, and VGG16 [4].

Recent works have shown that microarchitectures (e.g., SqueezeNet [5], ShuffleNet [6], EfficientNet [7], MobileNet [8]) with fewer parameters and small model sizes can achieve the same level of accuracy as the macroarchitectures (e.g., Inception [9], AlexNet [1], ResNet-50 [3], VGG16 [4]).

Modern object detection methods have shown excellent results in terms of accuracy and generalization. This is due to the complexity of the networks used as the models’ backbone. This complexity hinders their applications on edge computing devices that are usually liable to computational power and memory constraints. To deploy such models on these pieces of hardware while maintaining the performance (i.e., accuracy, robustness), it is necessary to optimize the models efficiently.

Different studies have proposed different compression methods, such as bit reduction, low-rank matrix decomposition, network pruning, sparsity, domain residual, knowledge distillation, etc. These methods have shown excellent results in terms of model size reduction, fast inference, and computational efficiency, without a significant decrease in accuracy when compared with the original model [5,10]. However, identifying the most effective and efficient methods based on the application requirements is challenging.

In this work, we propose a weighted score-based ranking scheme to address this problem. Our proposed scheme utilizes the performance metrics of the compressed models to evaluate and rank the compression methods. We show the effectiveness of this scheme by applying it on the baseline, compressed, and micro-CNN models trained on our dataset.

## 2. Project Description

### 2.1. Project Background

The research projects ROROGREEN and Smart Car Deck Solution (SCDS) are initiatives developed by DFDS, Denmark (Europe’s largest Ferry operator), and Tallink AS, Estonia (an Estonian based shipping company), respectively. These projects set out to develop an automatic vehicle detection (classification and localization), positioning, and tracking system for the cargo ships operated by both companies. The projects aim to digitize, automate, and optimize the end-to-end process of vessel stowage, loading/discharge of cargo units, and terminal operations. The actualization of these projects is crucial because it is envisioned that other shipping companies in Europe will also benefit from the projects’ results in the future.

### 2.2. Project Requirements

**Figure 1:**
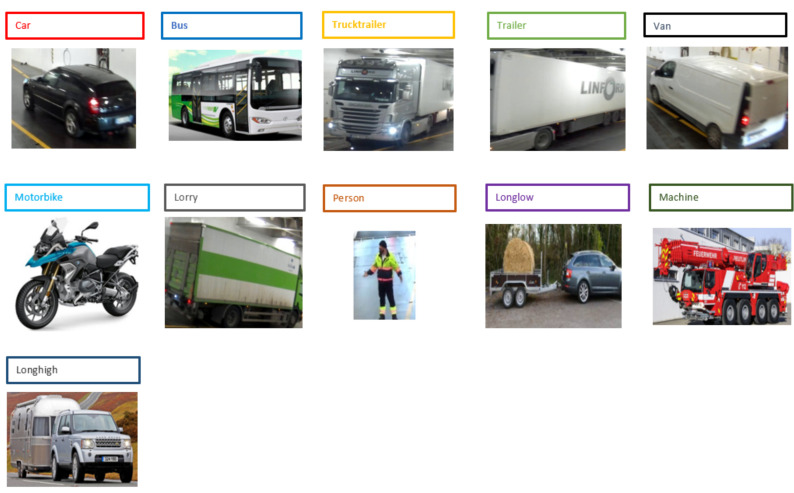
Sample of objects, including a person class, to be detected in the ROROGREEN and SCDS projects. There are eleven images and each corresponds to the respective target class.

The ROROGREEN and SCDS projects require a real-time automated solution that can track and monitor different objects on the decks of a cargo ship. Our goal is to achieve this using a cost-effective camera-based edge device that is capable of processing the images from the camera locally and in real-time. Our proposed solution is based on modern CNN-based object detection methods, which will be optimized for the proposed hardware. For the evaluation of the optimization methods implemented in this work, we used a portion of the entire dataset that consisted of 1400 images of different categories gathered locally. The sample of the dataset is described in Figure 1.

To fulfil the goal of the projects, we proposed a solution that leverages modern object detection methods. These methods serve as the backbone for the other functionalities of the system (e.g., tracking, positioning, monitoring). We extracted the requirements of the projects from the projects’ goals, use cases, and areas of use. We found that the requirements of both projects are quite similar. The ROROGREEN project aims to classify, localize, and track two classes of vehicles according to DFDS vehicle grouping. The SCDS project aims to detect and track ten classes of vehicles with people inclusive, which makes it a total of eleven classes. It is worth noting that DFDS’s vehicle classes are also embedded in Tallink’s vehicle categories.

The base networks of modern CNN-based object detection methods are usually deep. These usually require hardware having high processing power, high memory (flash size), and high RAM size. These requirements are usually lacking in resource-constrained devices; as such, deploying the models without optimization is impossible. Therefore, model compression is mandatory for efficient computation and storage, which, in turn, reduces the inference time without a significant drop in model accuracy. This will also minimize the overall power consumption of the system.

As discussed above, it is evident that our goal is to develop a real-time, low-power, cost-effective, and efficient solution using camera-based embedded hardware. This implies that the trained custom model must be optimized to meet the system/hardware requirements as highlighted below.

- High accuracy;
- Small model size;
- Small peak memory footprint;
- Fast inference;
- Computational efficiency.

## 3. Problem Statement

The ROROGREEN and SCDS projects aim to automate the loading and offloading operations of the cargo units by using the real-time data generated (e.g., vehicle type, object bounding coordinates, lane position, and other application parameters) by the camera-based embedded hardware installed on each deck. The generated data are processed by the external server, which automatically generates the loading/offloading plan, which is validated with the standard deck plan.

**Figure 2:**
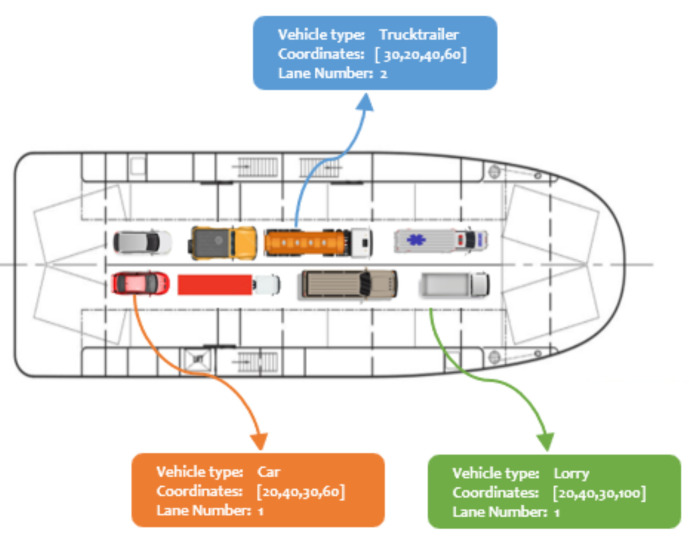
A contrived deck plan showing the vehicle loading operation. Each vehicle type occupies different regions and the respective lane position.

Predicting the vehicle type, bounding box coordinates, and position of each object requires modern object detection and position estimation algorithms. These methods must be energy-efficient, less computationally intensive, and accurate. This is quite onerous because of the choice of hardware required. The contrived visual representation of a ship deck containing different vehicle types on respective lanes is described in Figure 2.

**Figure 3:**
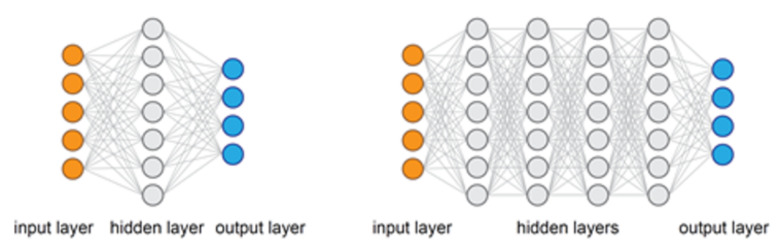
A visual representation of a deep neural network (**right**) and a shallow neural network (**left**).

We proposed a CNN-based object detection method. This was adopted due to the remarkable results it has shown. Modern object detection models are quite large in depth and sometimes width. The depth is usually characterized by the number of hidden layers in the backbone of the CNN architectures used. The depth contributes to the total number of parameters (i.e., the weights in each layer), which usually result in large models, as described in Figure 3. These models are power-inefficient, computationally expensive, and require large memory for storage. This cannot be processed by our low-power camera-based edge device. As such, the models need to be optimized/compressed for the hardware.

There are existing compression methods for large CNN models’ compression. Each method has its drawbacks and determining the most suitable and effective method is of major concern. To properly evaluate the effectiveness of each method, it is important to understand how the method affects the original models. Therefore, we can rank all the methods based on the following key parameters: model size, accuracy, memory footprint, computational cost, and inference time.

## 4. Related Work

CNN models have shown unprecedented results in solving problems related to computer vision (e.g., image classification, object detection, tracking). Due to the high computational power and memory that are required, this has impeded their adoption in embedded applications. This problem led to a new area of research (i.e., deep neural network compression) to tackle such challenges.

Several methods for model compression have been proposed in different studies. These methods have shown remarkable results, albeit with certain drawbacks. In this section, we categorize the methods into five groups: bit reduction, knowledge distillation, tensor decomposition, network pruning, and microarchitecture.

### 4.1. Bit Reduction

Bit reduction techniques have been around for quite a while [11,12]. These techniques aim to reduce the size of the model without a significant loss in the model performance. In practice, this is somewhat difficult to achieve due to the loss of information when approximating the 32-bit full precision weights and activations to a fixed point integer representation [13,14]. Quantization can be implemented using (16, 8, or 4 bits); however, there can be extreme cases where 2 bits or 1 bit are used to represent the weights and/or activations. These are referred to as binarization and ternarization. Binary networks encode the weights and activations with 1 bit (–1, 1), in contrast to ternary, which uses 2 bits (–1, 0, 1) [15].

The works of [11,12,16,17] showed the possibility of training deep networks with low bit-width weights. The weights and activations of the networks were discretized and a drastic reduction in model size with an insignificant drop in accuracy was achieved in [14]. An adaptive quantization framework that achieved a 20–40% higher compression rate, in contrast to non-adaptive quantization methods that relied on a uniform quantizer, was proposed in [18]. A vector-based quantization method that reduces the reconstruction in the network output was introduced in [19]. The work of [18] also showed that the network layers contribute differently to the model prediction result; therefore, it is logical not to use uniform bit-width quantization.

Quantization techniques have shown promising results for large model compression. This breakthrough has caused different industries developing on-device/edge-based artificial intelligence solutions to adopt the methods. It is worth noting that the lower the bit-width used in quantization, the higher the compression rate and the model sensitivity to accuracy degradation.

### 4.2. Knowledge Distillation

The idea of transferring the knowledge learnt by a large model to a small model is primordial. This has shown quite a reasonable result (i.e., accuracy). Ensembles (i.e., combining the predictions of several models) of large models were compressed using this method [20]. The authors used the parameters learned by the large, slow, and complex models to train an ensemble of small and fast models. This method gained traction from the results shown by the authors. The authors of [21] trained a single model by transferring the attributes of the ensemble of models to the single model. This achieved higher accuracy than the prediction of the individual model of the ensemble.

The transfer of knowledge from a large and accurate model does not guarantee that the small model will be accurate. It was demonstrated that not all the students (small models) can learn effectively from the teachers (large models) in [22]. The authors also pointed out that past work has not addressed this area but rather focused on the trend of the subject.

Knowledge distillation has proven to be very relevant in many applications. This is due to its simplicity and the ability to use synthetic data (data generated artificially) where real data are not readily available; however, the statistical attributes of the synthetic data must conform with the mimic real data [22].

### 4.3. Low-Rank Tensor Decomposition

Tensor decomposition is the generalization of low-rank matrix decomposition. Its use case has been extended to CNNs. CNN models are composed of different layers, which are defined by the types of mathematical operations performed in the layer. These layers include the convolutional layer (CL), activation layer (AL), fully connected layer (FCL), etc. A layer in the network is an array of nodes or neurons that can be expressed as a matrix or tensor (i.e., a generalized form of a matrix). Each node is a regression function that performs some computations (e.g., matrix multiplication) on higher-dimensional input data and a weights matrix. Matrix-based optimization techniques (e.g., singular value decomposition, eigendecomposition) can be applied to the convolutional and/or the fully connected layers [10,23] to reduce the number of parameters in these layers.

When a tensor (e.g., weights matrix) is factorized into its sub-components (i.e., sub-tensors or factors), all the sub-tensors do not contribute equally to the main tensor. This implies that the sub-tensors can be ranked based on the order of importance. An approximate tensor can be derived, which results in having a low rank in contrast to the original high-order tensor. Singular value decomposition (SVD) (the most commonly used matrix decomposition method) was used to compress the weights matrix in the fully connected layer to reduce the model size. A two-times increase was achieved in computation time by decomposing the weights in the convolutional layers in [24]. Kholiavchenko [25] proposed an iterative-based tensor decomposition technique and showed that a layer can be decomposed several times.

It is also worth pointing out that the compression ratio of the model when adopting this method is greatly dependent on the rank value (i.e., the most significant to the least significant). In the case of an extreme rank value, the model size will be drastically small and vice versa.

### 4.4. Network Pruning

It has been shown in several studies that deep neural networks are usually overparameterized. This is quite common in large and complex networks [26]. It affects the model convergence time and contributes to the computation and storage overheads. The goal of pruning is to reduce a large network to a smaller and faster network. This is possible because the parameters of the network do not contribute equally to the model output. As such, the level of contribution can be ranked by the order of importance and the less significant parameters can be pruned (i.e., set to zero). A penalty factor was introduced to the loss function to penalize the weights that do not contribute significantly to the network output, resulting in a smaller network, in [27]. A stochastic gradient descent (SGD) momentum-based pruning method for setting redundant weights to zero was proposed in [28].

The network parameters that can be pruned include the weights (connections), neurons (nodes), or convolutional filters (kernels). The choice of the parameters to be explored for pruning is dependent upon the application requirements (e.g., memory footprint, computational cost, bandwidth). A weights- or connections-only-based pruning technique is also referred to as unstructured pruning, in contrast to structured pruning, which involves the removal of low-rank neurons or filters [28]. Redundant weights were eliminated to compress the network size and compensate for the accuracy drop by retraining the network in [29].

Pruning is a very old compression method [30] and has shown its strength in the reduction of model size. The effectiveness varies with the techniques adopted (e.g., brute-force [30], penalty-factor [31], sensitivity error [32,33]). Network pruning usually results in an accuracy drop. When a network is over-pruned, it can render the network useless. This is why it is important to estimate the pruning threshold, evaluate the network after pruning, and retrain to compensate for the decrease in accuracy.

### 4.5. Microarchitecture

Microarchitecture is a concept for designing small and compact models. This method is based on the background information (i.e., residual knowledge) of the critical and most important blocks needed in the design of the CNN architecture. This is quite different from the other methods (i.e., pruning, quantization, binarization, tensor decomposition) because it does not rely on any external compression techniques that are usually applied to the CNN model either after or during training. It focuses on using domain knowledge to carefully design the network architecture. The same level of accuracy obtained by AlexNet [1] with 50-times fewer parameters was achieved in [5]. Before the term miroarchitecture was standardized by these authors [5], smaller kernel sizes (i.e., smaller-sized convolutional filters) were used in practice, and this has shown significant improvements in terms of model performance (i.e., speed, accuracy) [34,35,36].

The research on the design of efficient and lightweight CNN models has increased as a result of the exponential growth in the demand for real-time, efficient, and power-consumption-aware embedded computer vision applications in diverse areas. A category of models called MobileNet was proposed in [8]; these CNN microarchitectures have become some of the state-of-the-art methods for image classification and object detection for low-power and resource-constrained devices. Other models in this category are SqueezeNet [5], ShuffleNet [6], EfficientNet [7], and TinyYOLO [37].

## 5. Compression Methods, Evaluation, and Ranking

Our goal is to evaluate and develop a novel method for ranking the performance of state-of-the-art techniques for compressing deep learning models. The performance of each technique will be ranked using the following five key metrics: model size, accuracy, peak runtime memory usage, computational cost, and inference time. In this section, we describe each compression method and its implementation.

### 5.1. Baseline Model Description

This section describes the architecture of our baseline model. This simple and lightweight network was developed to serve as the reference model for evaluating the different compression methods for this work. The input layer of the network takes an RGB image of shape (64, 64, 3) and (3, 3) kernel filters were used throughout the entire network, resulting in a total of 1,106,209 parameters. The choice of the convolutional filter size was inspired by the work of [34]. The network includes a stack of two sets of CONV2D, RELU, BN, and POOL layers, followed by a set of CONV, RELU, and BN. The final block is the dense block, which consists of FC, BN, FC, and SOFTMAX layers, as shown in Table 1.

### 5.2. Compression Methods

CNNs play a vital role in deep learning methods for object detection. A CNN is composed of different layers. Each layer is made up of computational nodes (i.e., neurons) that process the input signals. The contribution made by each layer to the computation and memory requirements of the whole network is usually unevenly distributed. This is because of the different operations and parameters that are associated with each layer. The majority of the weights are in the dense layers (i.e., fully connected layer), but these account for a lesser percentage of the total floating-point operations. This implies that optimizing the FC layers alone will result in only a dramatic reduction in the model size, without a significant improvement in the overall speed, in contrast with optimizing the convolutional layers.

Several methods have been proposed for CNN model compression for resource-constrained devices. Each of these methods has its advantages and drawbacks. This makes it very challenging to identify the most appropriate, effective, and efficient compression method to adopt. The choice of the method is strictly dependent on the requirements of the application. In this section, we describe the compression methods that we considered in this work.

#### 5.2.1. Quantization

Bit precision reduction is an important concept in mathematics that has been widely adopted in different applications, including deep neural network compression. Quantization limits the width of the bit that is used to represent a digit or number. The bit width of the operands controls the precision level of the output when mathematical operations are performed.

**Figure 4:**
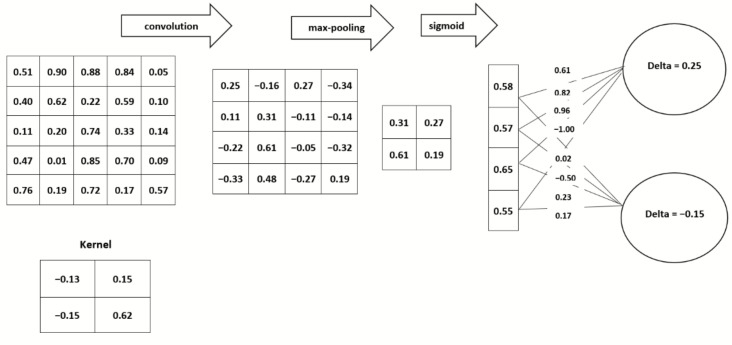
A CNN architecture with a normalized (5 × 5) input image convolved with a normalized filter (2 × 2 kernel) showing the three most common operations (convolution, pooling, and weights matrix multiplication operations) in a CNN. This low-level abstraction shows the internal computation performed on the network parameters (i.e., the input tensor (5 × 5 image), weights, and activations).

The most common types of operations that are performed in CNNs are convolution operation, bias addition, and the dot product of the weights matrix and float input tensor, as described in Figure 4. These operations are computed in a 32-bit full precision floating point. Quantization aims to replace the 32-bit floating-point operations with low-precision number formats such as 1 bit, 4 bit, 8 bit, or 16 bit. Binarization transforms full precision models into a single-bit model (the weights and activations are encoded using 1 bit). Binarization can be described as an extreme case of quantization where the weights and activations are encoded using 4 bit, 8 bit, or 16 bit.

A model can be quantized either during training (Bit-Reduction-Aware Training, also called Quantization-Aware Training) or after training (Post-Quantization). The latter often results in a significant decrease in accuracy. However, this can be mitigated by retraining the network to compensate for the decrease in accuracy as a result of the error induced during the quantization operation.

**Figure 5:**
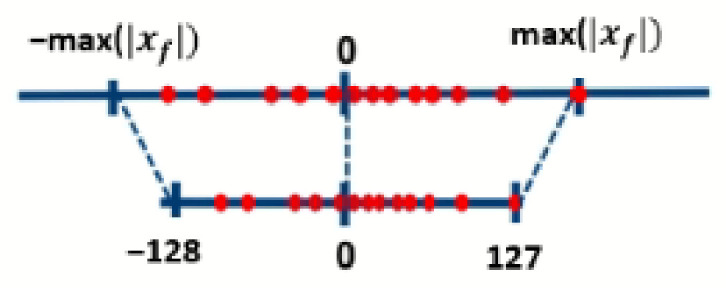
This figure shows the mapping of the input tensor float range to the 8-bit signed integer quantized range in symmetric mode.

We quantized the weights and activations of the baseline model using a symmetric mode 8-bit signed full integer quantizer implemented in Keras [38], using TensorFlow [39] as the computing engine. The mapping of the 32-bit input float tensors (i.e., weight matrix, activations) to the 8-bit quantized range is described in Figure 5. The mapping function (i.e., 8-bit quantizer) maps the input float tensors of the baseline model to the 8-bit quantized output. This function is defined in Equation (1):

(1) $$q_{8bit}=round\left(m_{f}i_{f}\right)$$

where $q_{8bit}$ is the 8-bit quantizer, $m_{f}$ is the multiplier, and $i_{f}$ is the input float tensor. The multiplier is the quantization constant that is multiplied with the float input tensor, as expressed in Equation (2):

(2) $$m_{f}=\frac{\frac{2^{7}-1}{2}}{\max(|i_{f}|)}$$

#### 5.2.2. Binarization

Binarization is a bit reduction technique that is considered an extreme case of quantization in which the weights and/or activations are encoded using a single bit (i.e., 1 bit). A single bit can be considered the atomic bit level of a number system; therefore, a significant decrease in the model accuracy is imminent due to loss of information during the binarization process. We did not binarize the first and last layer of the baseline model to minimize this loss.

In the binarization process, updating the weights during backward pass using the standard gradient descent approach is impossible because computing the loss gradient would result in zero in almost all conditions. We adopted a Straight-Through-Estimator (STE) pseudo function that has been proven to solve this issue.

We binarized the baseline model using Larq [40], an open-source Binary Neural Network library built on Keras [38]. The binarization function (a non-zero sign function) $b_{1bit}$ takes the float tensor as input and returns a binary output (−1 and +1), as shown in Equation (3):

(3) $$o_{1bit}=b_{1bit}\left(i_{f}\right),o_{1bit}\in \left\{-1,1\right\},i_{f}\in \left\{R\right\}$$

where $b_{1bit}$ is the binarization function, $o_{1bit}$ is the binary output generated, and $i_{f}$ is the input float tensor. During the backward pass, the loss gradient is calculated using the STE function, which takes the output tensors as input and returns a binary output, which is constrained to the threshold value, as expressed in Equation (4):

(4) $$loss_{gradient}=\left\{\begin{array}{cc} 1 & \mathrm{abs}(i_{f})\;\leq \;threshold_{value}\\ 0 & \mathrm{abs}(i_{f})\;>\;threshold_{value} \end{array}\right.$$

where the $threshold_{value}$ is the float value that controls the $loss_{gradient}$ and $i_{f}$ is the float tensor processed by the STE pseudo gradient function.

#### 5.2.3. Network Pruning

Pruning is one of the oldest methods for compressing large CNN models for low-power and resource-constrained devices. Pruning explores and exploits redundant parameters that do not contribute significantly to the model performance. The effectiveness of the pruning method is dependent on how efficiently we can evaluate the parameters that are redundant in the network.

Over-pruning the baseline model will decrease the accuracy and damage the network completely. This can be mitigated by evaluating the model based on certain criteria after each pruning operation in an iterative manner. There are different pruning methods (e.g., weight-only pruning, node-only pruning, or layer pruning).

In this work, we pruned the baseline model using the magnitude-based weight pruning approach as opposed to the neuron-based method. We implemented this pruning method because it does not affect (i.e., decrease) the model accuracy significantly, especially when the base model is not complex (i.e., having few hidden layers, as with the baseline model). The weights of the baseline model pruned were selected using rank-based criteria, calculated using the absolute value of the individual weight in Equation (5):

(5) $$rank_{weight}=\left|w_{i}\right|,w_{i}\in W$$

where $rank_{weight}$ is the rank of the individual weight, $w_{i}$ is the weight, and W is the weight matrix associated with each neuron within the respective layer of the network.

#### 5.2.4. Knowledge Distillation

Distilling knowledge (i.e., useful information) from a large model (the teacher) to a small model (the student) has shown excellent results in model compression. The concept of knowledge transfer is based on the idea that large models are robust and can learn complex patterns from data such that useful information can be transferred to the small model to mimic the behaviour of the large model.

We implemented the teacher-student model using a temperature-based softmax function at the output layer in Keras [38]. This technique was inspired by [21]. The teacher model (i.e., VGG16) was trained on our dataset, and the class probabilities vector for each data point (i.e., observation) was calculated and extracted. These probabilities vectors, also called soft labels, were distilled to the small model as the target label during training. We also trained the small model using the hard labels, and the overall losses generated by the small model were combined and weighted, as shown in Equation (6):

(6) $$\begin{array}{cc} \; & Loss_{total}=\alpha \times H\left(y,\sigma \left(z_{s}\right),T=1\right))+\beta \times \\ \; & H(\sigma \left(z_{t};T=t\right),\sigma \left(z_{s},T=t\right)) \end{array}$$

$Loss_{total}$ is the total loss, which is the combination of the student and distillation losses. The student loss is computed using the standard loss function by making the temperature parameter (*T* = 1). The temperature parameter controls the amount of information that can be distilled to the student. However, we need to keep in mind that the student has a threshold that limits the amount of information that it can retain from the teacher. The α and β are constants associated with the individual loss function taking the respective unnormalized log probabilities ($z_{s}$, $z_{t}$) for each class label.

#### 5.2.5. Tensor Train Decomposition

The decomposition of a matrix into its low-rank embedding is a very important concept in linear algebra. Matrix decomposition is used extensively in applied data processing for computation acceleration and data compression. Tensor decomposition is a means of generalizing the concept of low-rank matrix decomposition by treating the matrix as a tensor (i.e., a higher-order array). There are standard matrix decomposition methods (e.g., QR, LU, Eigen, singular value decomposition (SVD), etc). SVD is the most widely used method but cannot operate directly on higher-order data structures such as tensors. Working directly on tensors offers the benefit of keeping the correlation between data points intact.

Tensor decomposition is still relatively new. Few methods have been developed (e.g., Canonical Polyadic (CP), Tucker, Tensor-Ring, Tensor-Train, etc.). We adopted the TT decomposition method due to the computational time (i.e., reconstruction and decomposition time) and storage space advantages that it has over CP, Tucker, and other tensor decomposition methods.

TT decomposition factorizes a tensor into sub-tensors called cores/factors. The number of cores is dependent on the dimensions of the input tensor. TT is based on SVD and the factorized outputs are expressed as a train of tensors (i.e., a product of smaller core tensors). The dense layer in the baseline model has a set of weights in its nodes transformed into TT matrices (i.e., 4D tensor shape). The TT matrices are factorized into four TT cores and each element of the tensor can be reconstructed as defined in Equation (7):

(7) $$\begin{array}{cc} \; & T{(}_{i_{1},i_{2},i_{3},i_{4}})=\sum_{r_{1},r_{2},r_{3}}^{R_{1}R_{2}R_{3}}G^{1}\left(i_{1}r_{1}\right).\\ \; & G^{2}\left(r_{1}i_{2}r_{2}\right).G^{3}\left(r_{2}i_{3}i_{3}\right)G^{4}\left(r_{3}i_{4}\right) \end{array}$$

where *T* is the original tensor, $i_{n}$ represents the tensor indices, $r_{n}$ corresponds to the ranks of the tensor, $R_{i}$ are the compressed/contracted hidden indices, and $G^{i}$ are the TT cores.

We retrained the baseline model using TT, which transforms the input and output parameters of the dense layer, excluding the softmax layer, into TT matrices, and the outputs were decomposed into tensor cores. We implemented this method using the t3f framework [41], a tensor train library built on TensorFlow [39].

#### 5.2.6. Microarchitecture

The concept of designing sub-blocks/modules as a micro-unit in CNNs has shown promising results in terms of model size reduction and improved inference time without a significant decrease in model accuracy. The CNN microarchitecture relies on the residual knowledge that is adopted to carefully design each of the CNN sub-blocks that make up the entire network. The microarchitecture defines the dimensions and structure of each sub-module and how they are integrated to form the entire network.

There are several CNN microarchitectures that have been proposed for resource-constrained devices. In this work, we trained our dataset on MobileNet V1, MobileNet V2, MobileNet V3, and ShuffleNet. The results of these models were compared with those of the compressed models.

### 5.3. Evaluation and Ranking

An object detection model performs a detection task by fusing object classification and localization methods. Some methods involve using two separate algorithms (i.e., MobileNet and SSD). Other methods (e.g., YOLO) use a single model to perform both the classification and localization tasks. To optimize such a model for memory compression, speed, and accuracy, the parts of the model contributing to these metrics need to be optimized.

Much of the memory and computational power is expended by the base network of the object detection model. This formed the basis of our evaluation of the compression methods on the classification model instead of both the classification and localization models.

We based our evaluation and ranking on five key metrics: model size, accuracy, peak runtime memory footprint, computational cost, and inference time. The results corresponding to the key metrics obtained from the compressed models, micro-CNN models, and the base model are described in Table 2. These results were mean values calculated over a small number of experiments in order to reduce the error margin due to the stochastic nature of training CNN models.

We evaluated the baseline, compressed, and micro-CNN models on a Google Coral Development Board with the following technical specifications: Quad Cortex-A53 and Cortex-M4F processors, Edge TPU co-processor (supports only int8 operations), 1 GB LPDDR4 RAM, and 8 GB eMMC flash memory [42]. The performance (i.e., key metrics) of the compressed, micro-CNN, and base models was evaluated using the weighted score-based ranking scheme that we developed.

Our ranking scheme relies on two major components: the weights (i.e., relevance scores) assigned to the criteria (i.e., key metrics) and the computed scores of the results generated by the compressed models, base model, and micro-CNN models. It is critical that the weights and scores should have the same scale (i.e., value range). The raw results generated in Table 2 were not scaled and this does not meet the requirement of the weighted score-based ranking method.

We applied a scaling and scoring function to the unscaled results generated in Table 2. This function maps and scores each unscaled result to a value in the range (1–10). The scaling function is defined as:

(8) $$n_{scaled}=\frac{n-m_{min}}{m_{max}-m_{min}}\times \left(r_{max}-r_{min}\right)+r_{min}$$

where $n_{scaled}$ is the scaled output value, *n* is the metric value to be scaled into [$r_{min}$–$r_{max}$], $n_{min}$ is the minimum of the metric value range, and $n_{max}$ is the maximum of the metric value range. The $r_{min}$ and $r_{max}$ represent the minimum and maximum value of the target scale range (i.e., 1–10 as used in our experiment). The scale corresponds to the range of the weighted relevance score assigned to each evaluation metric.

Each metric was assigned a weighted relevance score in the range (1–10); a high weighted relevance score (e.g., 10) indicates that the corresponding metric has the highest priority (i.e., most significant) and vice versa, as shown in Table 3. The scores (i.e., the scaled metric values) and weights assigned to the evaluation metrics are described in Table 3.

The weighted relevance score controls the significance score of a metric during the evaluation of the performance of the compressed models, base model, and micro-CNN models. The weight of each metric is determined based on the application requirements. We assigned a weight value to the individual metric, as shown in Table 3. These values were generated based on our application requirements. The values can be adjusted to suit other applications.

The scaled values were scored on a scale of (1–10). A score of 10 assigned to the model per metric value means that the model with respect to the metric is the most significant, while a score of 1 means that the model is less significant. Table 4 shows the score of each model per metric value computed from the results (scaled metric values) obtained in Table 3.

The weighted score was calculated by computing the product of the corresponding weight of the relative importance score assigned to the metric and the score of the model corresponding to the metric. The mean weighted scores of the compressed models, micro-CNN models, and base model were ranked using an inverse ranking method similar to Spearman’s rank approach (i.e., the largest mean weighted score was assigned a rank of 1, the second-largest mean weighted score was assigned a rank of 2, and as the mean weighted score decreases, the rank number increases by 1 until the maximum rank number n is reached, where n is the count of the models evaluated), as shown in Table 5.

We calculated the mean weighted score per model for all metrics by computing the ratio of the arithmetic mean of the weighted scores and the sum of the weights assigned to all metrics. The equation is defined as:

(9) $$W=\frac{\sum_{i=1}^{n}w_{i}m_{i}}{\sum_{i=1}^{n}w_{i}}$$

where *W* is the mean weighted score, *i* is the metric index, *n* is the total number of evaluation metrics, $w_{i}$ is the weighted relevance score assigned to each metric, and $m_{i}$ is the calculated score of the model corresponding to the metric.

**Figure 6:**
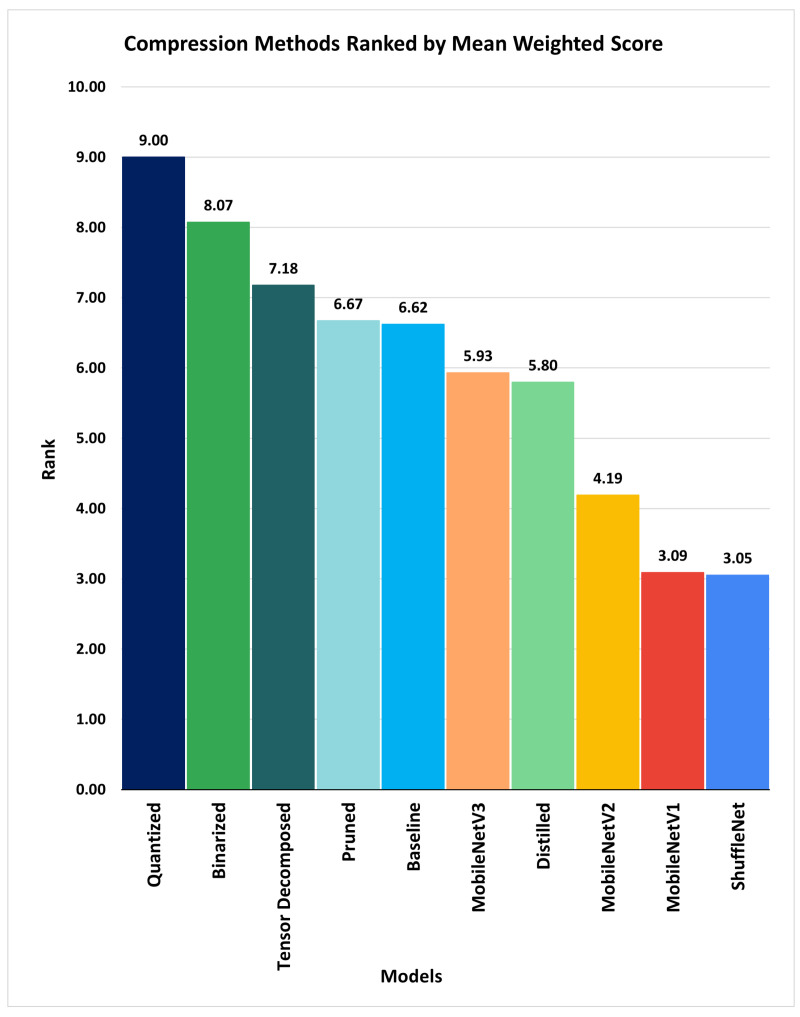
Compression methods ranked by the mean weighted score.

The ranking result showed that the application’s most effective, efficient, and suitable compression method is quantization with the rank of 1 (having the largest mean weighted score of 9.0), followed by binarization (having a mean weighted score of 8.07), as shown in Figure 6. As the rank number increases, the methods’ effectiveness, efficiency, and suitability to meet the application requirements decrease.

## 6. Discussion

The state-of-the-art methods for compressing deep learning models have shown excellent results in terms of peak runtime memory reduction, low latency, model size reduction, and computational efficiency, without a significant decrease in accuracy. These results vary from one compression method to another. The application requirements also differ, thus making it challenging to choose the optimal compression method for different applications (e.g., our ROROGREEN and SCDS projects). To address this, we propose a weighted score-based ranking scheme that enables us to evaluate and rank the compression methods based on their computed weighted mean scores. The weighted mean score is dependent on the metric scores and the weights assigned to the evaluation criteria, as shown in Table 4. The values in Table 4 correspond to the metric scores computed for each scaled evaluation criterion with respect to all the models evaluated.

The weight assigned to each evaluation metric determines the relevance score of the individual criterion. A high relevance score assigned to the metric with respect to the score range gives the metric a high effect when calculating the weighted metric score and the weighted mean score, as shown in Table 5. The metric scores correspond to the scaled metric values in Table 3. The scaled metric values correspond to the scaled model results in Table 2. The scaling ensures that all the values (i.e., the results obtained for all the metrics during model evaluation) use a common range. This eliminates the dominance effect of larger values regardless of the units when calculating the weighted mean scores. The metric scores ensure that the least and the most significant values of the evaluation metrics have the same interpretation (e.g., the lower the model size, the higher the compression rate with respect to the original model, whereas the higher the accuracy, the better).

We compare and rank the result of the computed weighted mean score of the baseline, compressed (i.e., quantized, binarized, pruned, distilled, and tensor-trained), and micro-CNN (i.e. MobileNetV1, MobileNetV2, MobileNetV3, and ShuffleNet) models, as shown in Figure 6. The weighted mean score for each model indicates the suitability score of the compression method that produces the model. A higher suitability score is desirable. The higher the weighted mean score (i.e., suitability score), the higher the rank (the highest rank is equivalent to 1 and it decreases as the rank value increases). The model with the highest weighted mean score (i.e., a rank of 1) is considered the most efficient and effective model for the hardware. As such, the compression technique that produces the model is considered the most suitable compression method for optimizing the base model for the application.

The limitation of our proposed scheme is the selection of weights for the performance metrics. These weights must reflect the priorities (i.e., level of significance) of the application criteria. This is considered a limiting factor because the weights are dependent on the application requirements, such as the available hardware resources (e.g., processor capability, flash size, RAM, etc.), model requirements, and the other requirements that are specific to the application (e.g., real-time capability, on-device model performance, etc.), and thus must be chosen appropriately. We do not consider this a major limiting factor because we have demonstrated how we selected the weights that are appropriate for the performance metrics in our case studies (ROROGREEN and SCDC projects).

Choosing the optimal model compression method for on-device AI applications is challenging due to the lack of an application-specific framework for evaluating methods for deep learning model compression for resource-constrained edge devices. We addressed this issue using our proposed weighted score-based ranking scheme. The scheme helps us to identify the quantization technique as the optimal method for compressing our object detection and tracking models for the application based on our requirements.

## 7. Conclusions and Future Work

In this work, we evaluated and ranked the state-of-the-art methods for CNN model compression for resource-constrained edge devices using a weighted score-based ranking scheme that we developed. Our ranking method uses five key metrics (i.e., model size, accuracy, inference time, computational cost, and peak memory footprint) computed for each model generated by the compression methods. We introduced an individual weight to these key metrics. The individual weight reflects how relevant/important the metric is in our application.

This work was motivated by the lack of a clear framework/method for selecting the most efficient methods for compressing CNN models for edge computing devices (e.g., micro-controllers, small computer boards, portable devices, mobile devices, DNN hardware accelerators, etc.). As such, we developed a weighted score-based ranking scheme to address this issue.

We applied our method to the baseline model developed, compressed versions of the baseline model produced by the state-of-the-art compression methods that we implemented, and the micro CNN models trained on a portion of the SCDS and ROROGREEN dataset. According to the ranking of the mean weighted scores computed, the quantized model obtained the highest rank, with a mean weighted score of 9.00, followed by the binarized, model having a mean weighted score of 8.07. ShuffleNet has the lowest rank in Table 5, with a mean weighted score of 3.05. This clearly shows that the quantization technique is the most suitable model compression method for both the SCDS and ROROGREEN projects.

Determining the most effective, efficient, and optimal method/methods for optimizing deep learning models for edge devices can be very challenging. We addressed this issue using our weighted score-based evaluation and ranking method.

In the future work, we will focus on how we can further improve the metrics of the best-ranked method (i.e., the quantized model).

## Figures and Tables

**Table 1:** A table showing a summary of all the layers of the baseline model architecture.

Layer Type	Output Size	Parameters
CONV2D	(None, 64, 64, 32)	864
BN	(None, 64, 64, 32)	96
MAXPOOL2D	(None, 32, 32, 32)	0
CONV2D	(None, 32, 32, 64)	18,432
BN	(None, 32, 32, 64)	192
MAXPOOL2D	(None, 16, 16, 64)	0
CONV2D	(None, 16, 16, 64)	36,928
BN	(None, 16, 16, 64)	192
FLATTEN	(None, 16384)	0
DENSE	(None, 64)	1,048,576
BN	(None, 64)	192
DENSE1	(None, 11)	704
ACTIVATION	(None, 11)	0
	TOTAL PARAMETERS	1,106,209

**Table 2:** Evaluation metrics results of the compressed models, base model, and micro-CNN models.

	Uncompressed	Compressed					Micro-CNN			
**Metric**	**Baseline**	**Quantized**	**Binarized**	**Pruned**	**Distilled**	**Tensor-Trained**	**V1**	**V2**	**V3**	**ShuffleNet**
Model Size (KB)	4429.61	1119.54	105.50	1308.82	4429.61	258.02	12,856.38	8935.84	12,171.81	7761.17
Accuracy (%)	77.23	76.95	67.10	74.64	72.04	71.47	68.88	64.43	71.18	65.99
Inference Time (ms)	22.88	13.65	5.40	22.64	22.87	17.31	39.09	20.79	16.79	40.09
Computational Cost (MFLOPs)	66.44	7.29	6.96	66.44	66.44	64.05	94.01	52.88	15.24	158.63
Peak Memory Footprint (KB)	8907.81	3705.47	1775.78	8900.78	8907.59	4971.88	19,857.04	12,582.8	10,550.02	12,796.72

**Table 3:** Weighted relevance score assigned to evaluation criteria and model results scaled to (1–10), which corresponds to evaluation metrics’ scale range.

		Uncompressed	Compressed					Micro-CNN			
**Metric**	**Weight**	**Baseline**	**Quantized**	**Binarized**	**Pruned**	**Distilled**	**Tensor-Trained**	**V1**	**V2**	**V3**	**ShuffleNet**
Model Size (KB)	8	4.05	1.72	1.0	1.85	4.05	1.11	10.00	7.23	9.52	6.40
Accuracy (%)	10	10.00	9.80	2.88	8.18	6.35	5.95	4.13	1.00	5.75	2.10
Inference Time (ms)	6	4.87	2.82	1.00	4.81	4.86	3.63	8.45	4.40	3.52	10.00
Computational Cost (MFLOPs)	6	4.53	1.02	1.00	4.53	4.53	4.39	6.17	3.72	1.49	10.00
Peak Memory Footprint (KB)	7	4.55	1.96	1.00	4.55	4.55	2.59	10.00	6.38	5.37	6.49

**Table 4:** Metric scores corresponding to the scaled metric value.

		Uncompressed	Compressed					Micro-CNN			
**Metric**	**Weight**	**Baseline**	**Quantized**	**Binarized**	**Pruned**	**Distilled**	**Tensor-Trained**	**V1**	**V2**	**V3**	**ShuffleNet**
Model Size (KB)	8	6.00	8.00	10.00	7.00	6.00	9.00	2.00	4.00	3.00	5.00
Accuracy (%)	10	10.00	9.80	2.88	8.18	6.35	5.95	4.13	1.00	5.75	2.10
Inference Time (ms)	6	3.00	9.00	10.00	5.00	4.00	7.00	2.00	6.00	8.00	1.00
Computational Cost (MFLOPs)	6	5.00	9.00	10.00	5.00	5.00	6.00	4.00	7.00	8.00	3.00
Peak Memory Footprint (KB)	7	7.00	9.00	10.00	7.00	7.00	8.00	3.00	5.00	6.00	4.00

**Table 5:** Compressed models, base model, and micro-CNN models ranked by the weighted mean score.

		Uncompressed	Compressed					Micro-CNN			
**Metric**	**Weight**	**Baseline**	**Quantized**	**Binarized**	**Pruned**	**Distilled**	**Tensor-Trained**	**V1**	**V2**	**V3**	**ShuffleNet**
Model Size (KB)	8	48.00	64.00	80.00	56.00	48.00	72.00	16.00	32.00	24.00	40.00
Accuracy (%)	10	100.00	98.00	28.80	81.80	63.50	59.50	41.30	10.00	57.50	21.00
Inference Time (ms)	6	18.00	54.00	60.00	30.00	24.00	42.00	12.00	36.00	48.00	6.00
Computational Cost (MFLOPs)	6	30.00	54.00	60.00	30.00	30.00	36.00	24.00	42.00	48.00	18.00
Peak Memory Footprint (KB)	7	49.00	63.00	70.00	49.00	49.00	56.00	21.00	35.00	42.00	28.00
Weighted Mean Score		6.62	9.00	8.07	6.67	5.80	7.18	3.09	4.19	5.93	3.05
Rank		5	1	2	4	7	3	9	8	6	10

## Data Availability

The ROROGREEN and SCDC datasets used are not publicly available.

## Abbreviations

The following abbreviations are used in this manuscript:

AL	Activation Layer
BN	Batch Normalization
CNN	Convolutional Neural Network
CP	Canonical Polyadic
Conv2D	Convolution 2D
DNN	Deep Neural Network
F-RCC	Faster Region-Based Convolutional Neural Network
FC	Fully Connected
FCL	Fully Connected Layer
ReLU	Rectified Linear Unit
SVD	Singular Value Decomposition
SGD	Stochastic Gradient Descent
TT	Tensor Train
